# T-Cell Repertoire in Tumor Radiation: The Emerging Frontier as a Radiotherapy Biomarker

**DOI:** 10.3390/cancers14112674

**Published:** 2022-05-27

**Authors:** Constantin N. Baxevanis, Angelos D. Gritzapis, Ioannis F. Voutsas, Panagiota Batsaki, Maria Goulielmaki, Maria Adamaki, Vassilios Zoumpourlis, Sotirios P. Fortis

**Affiliations:** 1 Cancer Immunology and Immunotherapy Center, Saint Savas Cancer Hospital, 11522 Athens, Greece; baxevanis@ciic.gr (C.N.B.); agkritzapis@agsavvas-hosp.gr (A.D.G.); ivoutsas@agsavvas-hosp.gr (I.F.V.); pmpatsaki@agsavvas-hosp.gr (P.B.); mgoulielmaki@eie.gr (M.G.); 2 Biomedical Applications Unit, Institute of Chemical Biology, National Hellenic Research Foundation (NHRF), 11635 Athens, Greece; madamaki@eie.gr (M.A.); vzub@eie.gr (V.Z.)

**Keywords:** radiotherapy, biomarkers, immunotherapy, cancer, abscopal effect, TCR repertoire

## Abstract

**Simple Summary:**

Radiotherapy constitutes an essential component of the treatment for malignant disease. Besides its direct effect on cancer cells, namely, DNA damage and cell death, ionizing irradiation also mediates indirect antitumor effects that are mostly mediated by the immune system. Investigations into the processes underlying the interaction between radiotherapy and the immune system have uncovered mechanisms that can be exploited to promote the antitumor efficacy of radiotherapy both locally in the irradiated primary tumor and also at distant lesions in non-irradiated tumors. Because of its capacity to stimulate antitumor immunity, radiotherapy is also applied in combination with immune-checkpoint-inhibition-based immunotherapy. This review discusses the important pathways that govern the synergistic interactions between ionizing radiation and antitumor immune reactivity. Unravelling these involved mechanisms is mandatory for the successful application of anticancer radiotherapy and immunotherapy. We also place emphasis on the need for biomarkers that will aid in the selection of patients most likely to benefit from such combined treatments.

**Abstract:**

Radiotherapy (RT) is a therapeutic modality that aims to eliminate malignant cells through the induction of DNA damage in the irradiated tumor site. In addition to its cytotoxic properties, RT also induces mechanisms that result in the promotion of antitumor immunity both locally within the irradiation field but also at distant tumor lesions, a phenomenon that is known as the “abscopal” effect. Because the immune system is capable of sensing the effects of RT, several treatment protocols have been assessing the synergistic role of radiotherapy combined with immunotherapy, collectively referred to as radioimmunotherapy. Herein, we discuss mechanistic insights underlying RT-based immunomodulation, which also enhance our understanding of how RT regulates antitumor T-cell-mediated immunity. Such knowledge is essential for the discovery of predictive biomarkers and for the improvement of clinical trials investigating the efficacy of radio-immunotherapeutic modalities in cancer patients.

## 1. Introduction

Radiotherapy (RT) constitutes an essential component of the therapeutic regime that is applied to cancer patients with solid tumors. Two main types of RT are performed in clinical practice: external beam and internal RT. These mainly differ in the way in which radiation is delivered to the tumor site. External beam radiation (EBR) is delivered locally to the tumor by an outer source in the form of high-energy rays (photons, protons, or electrons). On the contrary, internal radiation is performed through the introduction of radioactive sources to the patient’s body and can act either locally (brachytherapy) or systemically (systemic therapy) [1]. The method of choice is largely dependent on several parameters, including type, size, and location of the tumor, as well as phenotypic characteristics of the patient, e.g., age and potential comorbidities. Regarding EBR, which is the most common type of RT, the location and radiosensitivity of the tumor within the body defines the energy levels that are reached during the application of RT. Accordingly, stereotactic RT can deliver very high energy doses to strictly delineated sites, thus facilitating the treatment of small and deep tumors [2]. Hypofractionated radiation, i.e., several daily doses of about 2 Gy that sum up to a total dose of 45–50 Gy, has prevailed as a “standard” approach for the confinement of localized disease, after surgical resection of the tumor. Nonetheless, while total doses of maximum 45 Gy are efficient against most cancer types, higher radiation fractions might be necessary for the treatment of radioresistant tumors, such as melanoma [3]. In addition to the direct cytotoxic effects that are mediated via tumor cell DNA damage, RT exerts antitumor activity via enhancement of antitumor immunity both locally, within the tumor microenvironment (TME), and in the periphery, as an abscopal effect, resulting in the regression of tumors at distant sites [4,5]. The latter is believed to be mediated via RT-induced immune-dependent mechanisms, in the sense that the dying tumor cells release antigens that are taken up by professional antigen-presenting cells (i.e., dendritic cells, DC) and activate T lymphocytes in the draining lymph nodes, thus potentiating antitumor immune responses [5]. RT also induces the release of proinflammatory cytokines and damage-associated molecular patterns (DAMPs), which result in an overall activation of the immune system [6,7]. Considering the growing interest in clinical research for more effective cancer immunotherapies, along with the capacity of RT to enhance antitumor immunity, the combination of RT with immunotherapies is emerging as a promising therapeutic modality in the field of cancer treatment [8]. This review aims to provide an update on the role of RT in the activation of the antitumor immune machinery and to discuss the potential of radioimmunotherapy to significantly improve clinical applications for cancer treatment.

## 2. Radiotherapy and the Immune Response

The inflammatory machinery in the TME, comprising a plethora of cytokines with the capacity to either promote or inhibit antitumor immune reactivity, is of paramount importance for tumor development and progression [9]. RT alters this inflammatory milieux by modulating the cytokine signaling machinery in such a way so as to result in the maturation of antigen-presenting cells (APCs) and in the recruitment of cytotoxic T lymphocytes (CTLs) to the TME [9]. Hallahan et al. were the first to provide such evidence by demonstrating that irradiated, cultured human sarcoma cells are characterized by increased expression levels of the TNF-α gene, with subsequent TNF-α protein production [10]. Burnette et al. later reported that application of ablative RT on murine tumors generates tumor destructive innate and adaptive immune pathways that are dependent on the production of type I interferons (mainly IFN-α/β) within the TME [11]. Further experiments performed mainly in tumor-bearing mice demonstrated that RT induced IFN-γ production within the primary tumor, which was followed by increased intratumoral T-cell trafficking through increased expression of adhesion molecules by endothelial cells (including intercellular adhesion molecule-1, vascular cell adhesion molecule-1, and E selectin) and in the presence of high levels of T-cell chemoattractants [9,11,12,13]. These observations suggest that the effects of ionizing radiation are not restricted to T cells but also expand to the TME, with the regulation of mechanisms resulting in tumor cell eradication. Such mechanisms mostly constitute immunological pathways that are principally controlled by the irradiation-activated nuclear factor κB (NF-κB) and reactive oxygen species (ROS)-related downstream signaling [14]. NF-κB activation and translocation to the nucleus induces the expression of proinflammatory genes including interleukin-1 (IL-1), IL-6, IL-10, and tumor necrosis factor-α (TNF-α), among others [15,16]. ROS also affect signaling pathways regulated by TNF and additionally activate NF-κB, further enhancing TNF production [17].

**Figure 1:**
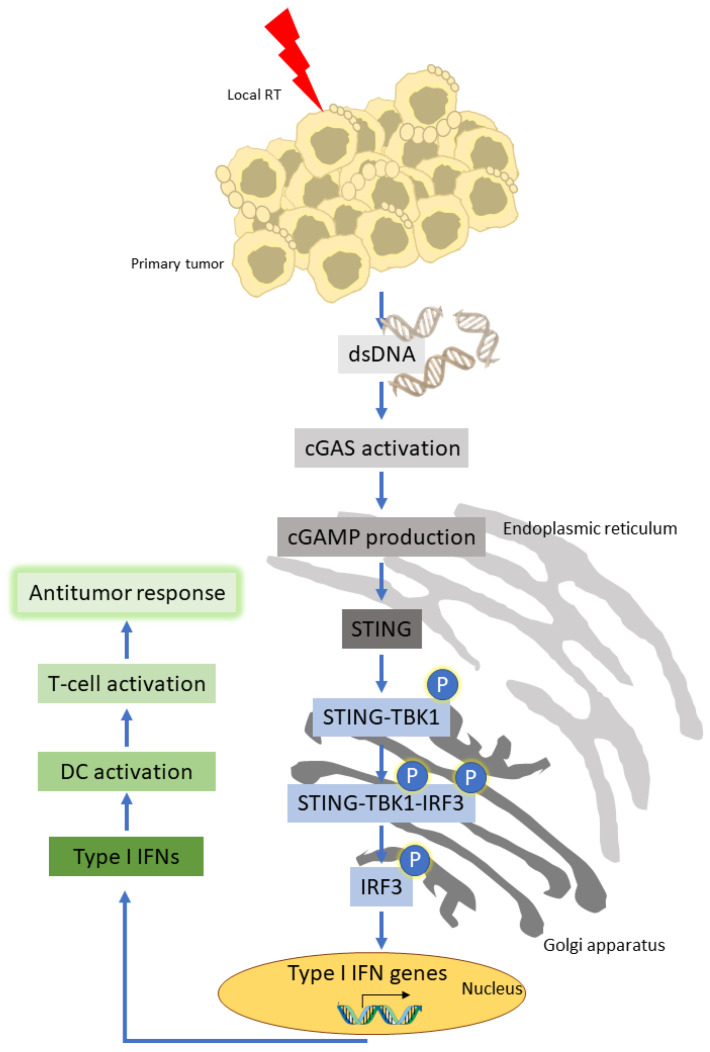
RT-mediated STING pathway activation results in the initiation of the antitumor immune response. Schematic initiation of immune responses to RT-mediated tumor DNA damage takes place in the presence of the DNA sensor cyclic GMP-AMP synthase (cGAS) that detects double-stranded DNA (dsDNA) in the cytosol. Following cGAMP synthesis by the activated cGAS, STING is also activated by direct binding to cGAMP. STING then translocates from the endoplasmic reticulum to the Golgi apparatus where it binds to the TANK-binding kinase 1 (TBK1) and initiates signal phosphorylation processes that result in the phosphorylation and activation of the transcription factor IRF3. After an initial binding of IRF3 to the STING–TBK1 complex, IRF3 dissociates and translocates to the nucleus where it induces type I IFN production. Type I IFN (mainly IFN-α/β) signaling leads to activation of DC, which in turn activate CD8+ T cells (via presentation of tumor antigens released by the dying tumor cells) to mediate specific antitumor immune responses.

Further evidence supporting the modulation of antitumor immunity by irradiation is provided by the DNA damage, which is induced in the irradiated tumor cells, resulting in immunogenic cell death [18,19]. Specifically, it has been shown that both in vitro and in vivo exposure of irradiated tumor cells to calreticulin, followed by the release of high mobility group protein B1 (HMGB1), has an immunoadjuvant effect that leads to DC maturation via toll-like receptor 4 (TLR4) signaling, uptake of the released tumor peptides, and cross-presentation by DC [20]. Other studies have indicated a prominent role for the host STING pathway, a pathway that senses DNA from irradiated tumor cells and induces various downstream activating signaling pathways, including the activation of IRF3 and subsequently IFN-β gene expression. Components of the STING signaling pathway are modulated by multiple post-translational alterations and are closely connected with cellular processes that have a significant impact on cancer immunotherapy. In particular, the direct DNA damage by RT can induce innate immune responses via cytosolic DNA sensors that detect double-stranded DNA. The cyclic GMP-AMP (cGAMP) synthase (cGAS) belongs to the key DNA sensors and, upon direct binding to the DNA, generates the cyclic dinucleotide 2′–5′ cGAMP; this in turn activates the stimulator of interferon genes (STING), a transmembrane protein in the endoplasmic reticulum (ER) [21,22]. The activated STING translocates to the Golgi apparatus where it binds to the TANK-binding kinase 1 (TBK1), initiating the phosphorylation processes for the trimolecular complex STING–TBK1–interferon regulatory factor 3 (IRF3), which in turn induces the expression of type I IFNs [23,24,25]. By performing experiments in tumor-bearing mice, Woo et al. also reported that STING-mediated expression of IFN-β by DC in the TME is regulated by tumor-associated antigens capable of triggering activation of CD8+ T cells [26] (Figure 1). Thus, optimal activation of STING is a necessary prerequisite for optimal type I IFN production (IFN-α/β), maturation, and activation of DC; the latter is followed by efficient priming of CD8+ CTLs specifically recognizing tumor peptides expressed by tumor cells via MHC class I molecules [27]. Moreover, direct induction of type I IFNs by irradiation increases endogenous tumor cell CXCR3 chemokine levels, which attract CTLs to the TME [28]. In addition, because IFN-γ is locally secreted in response to irradiation [29,30], such CTLs may undergo further activation in irradiated tissue with improved antitumor cytotoxic activity. Last but not least, ionizing irradiation has been reported to induce or enhance expression of MHC class I molecules by tumor cells in vitro and to expand the intracellular tumor peptide pool, thereby generating unique MHC-class-I-binding peptides further potentiating antitumor immunity [31].

Overall, RT appears to affect the intrinsic reactions of the immune system in the TME via modulation of cytokine production, maturation, and activation of DC, as well as through the triggering of T-cell infiltration and activation. In this scenario, CD8+ T cells, IFN-γ- and IFN-α/β-producing cells, and the detection of tumor-cell-derived danger signals through the TLR4 receptor on DC, followed by induction of CTLs, constitute essential players in radiation-induced antitumor immune responses. These observations suggest that RT has the potential to shape a more immunogenic TME that may provide the ground for more efficient immunotherapies and for substantial improvement of clinical responses in cancer patients. 

## 3. Abscopal Effects

**Figure 2:**
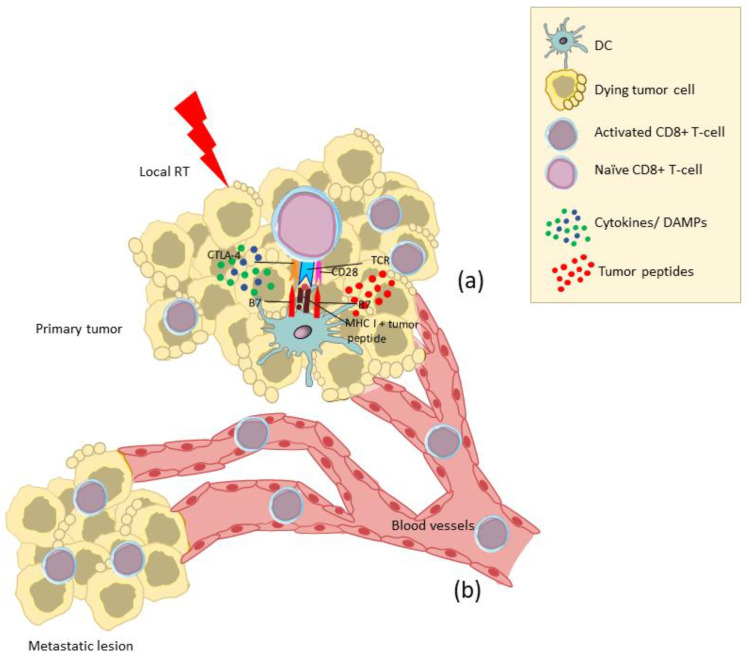
The abscopal effect (I). The cellular stress induced by local RT in the primary tumor will lead to the generation of an inflammatory milieux consisting of cytokines and DAMPs and to the release of tumor peptides. DC loaded with tumor peptides will cross-present them to naïve CD8+ T cells within the TME inducing their activation (**a**). The activated CD8+ T cells will mediate local (**a**) and distant (**b**) tumor cytotoxicity. RT, radiotherapy; DAMPs, damage-associated molecular patterns; DC, dendritic cell; TME, tumor microenvironment; MHC I, major histocompatibility complex class I.

**Figure 3:**
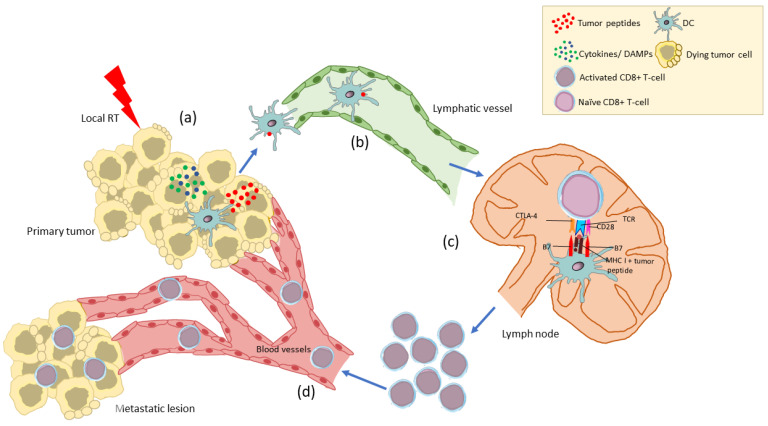
The abscopal effect (II). The DC loaded with tumor peptides in the inflammatory TME (**a**) travel via lymphatic vessels (**b**) to the lymph node to prime naïve CD8+ T cells (**c**). The activated CD8+ T cells enter the circulation via blood vessels (**d**) and attack both the primary tumor and non-irradiated metastatic lesions. RT, radiotherapy; DAMP, damage-associated molecular pattern; DC, dendritic cell; TME, tumor microenvironment; MHC I, major histocompatibility complex class I; TCR, T-cell receptor.

Immune lymphocytes and DC activated within the TME via the RT-induced immunomodulation may spread out via lymphatic and blood vessels and mediate antitumor immunity at distant sites. This abscopal effect is at least partially mediated by TME-derived T cells that have been activated upon recognition of tumor antigens released by the irradiated dying tumor cells and presented to them by DC within the inflammatory milieux of TME mainly consisting of cytokines and DAMPs [7,32] (Figure 2). Alternatively, DC loaded with tumor peptides in the irradiated TME may cross-present these peptides to naïve T cells in the lymph nodes [32] (Figure 3). In this respect, the abscopal effect may be considered as the result of RT acting as an in situ tumor vaccine. 

Pioneer work in this field by Demaria et al. has demonstrated that a single fraction of 2 Gy combined with fms-like tyrosine kinase 3-ligand (Flt3-L)-induced local and systemic T-cell-dependent antitumor immunity results in the rejection of a mammary carcinoma within the radiation field and at distant sites in syngeneic tumor-bearing mice [5]. In another study, it was shown that local radiation induced functional tumor-specific CTLs with potent antitumor activity, which was, however, abrogated when tumor-bearing mice were treated with anti-CD8 mAb [33]. Importantly, the same study also demonstrated that tumor-draining lymph nodes played an essential role in activating tumor-specific CTLs and that the combination of RT with Th1 cell therapy had a more profound antitumor systemic effect with the generation of immunological memory [33]. These studies have provided enough evidence to suggest that the abscopal effect is significantly more potent when combined with therapies that aim to activate the immune system. 

Considering that the abscopal effect depends on the actions of RT to mediate a shift from an immune-suppressive TME towards a more immunogenic TME, then it can be easily understood that treatments that supply the TME with immune-activating agents would constitute the most suitable candidates for combination therapy with local RT in order to generate robust abscopal effects. Several combinations of RT with immunomodulators (including vaccines and immune checkpoint inhibitors) have been tested for their capacity to induce local and systemic antitumor activity in preclinical mouse/tumor models [3,34]. Some of these combination regimens have been implemented in clinical trials, however, with limited success [7,34,35]. This might have been due to one or several limitations that are associated with the implementation of immunotherapy into clinical practice such as the tumor mutational burden, tumor heterogeneity in different metastatic sites, suppressor circuits within the TME, and expression of immune checkpoints, as well as the dosage and frequency of RT [34]. Experimental models may be useful for investigating the mechanisms underlying such negative results by focusing on the analysis of the above components and for conducting improved therapeutic combination strategies. The application of reverse translational research may help to identify the biomarkers that accurately predict the effective generation of abscopal effects so as to select the patients most likely to benefit from combination treatments of RT with immunotherapies.

## 4. The Immunosuppressive Effects of RT in the TME

Apart from the capacity to stimulate anti-tumor immunity, RT may also confer immunosuppressive properties to the TME, and this is not only evident as immune evasion and a continuity in tumor growth, but also as an absence of abscopal effects at distant metastatic sites [36]. This dual ability to either enhance or suppress anti-tumor immunity seems to be dependent upon the dose and fractionation schedule of RT, with lower doses and increased number of fractions being associated with an immune suppressive phenotype [37,38]. In this context, RT has been shown to promote the emergence of a variety of immunosuppressive factors within the TME, including but not limited to immunosuppressive cytokines, immune checkpoint molecules, and suppressive immune cell types [38]. 

In the TME, low-dose RT causes the upregulation of colony-stimulating factor 1 (CSF1), a growth factor that is responsible for the differentiation, recruitment, and immunosuppressive properties of tumor-associated macrophages (TAMs) and myeloid-derived suppressor cells (MDSCs) [39,40,41,42]. The activated TAMs and MDSCs secrete tumor growth factor-β (TGF-β), among other cytokines, which negatively regulates the anti-tumor immune response using various mechanisms, such as conversion of naïve CD4+ T cells into Tregs; activation of Tregs; and inhibition of effector T-cell differentiation, maturation, and activation [36,38]. In addition to CSF1 and TGF-β, RT causes tumor cells to overexpress the C-C motif ligand 2 (CCL2) chemokine, which in turn induces the recruitment of inflammatory macrophages expressing the CCL2 receptor (CCR2) to the tumor site; tumor cell recognition and uptake by inflammatory macrophages release anti-inflammatory signals that facilitate tumor tolerance and therefore contribute to the signaling inhibition of effective anti-tumor immune responses [36,43]. These observations suggest that CCL2/CCR2 inhibition may improve therapeutic responses to RT, and for this reason several such antagonists are currently being investigated in clinical trials, both alone and in combination with other forms of therapy, such as immune checkpoint blockade [44,45,46]. 

RT exacerbates the hypoxic stress by inducing upregulation of hypoxia-inducible factor-1α (HIF-1α), a key transcription factor of hypoxia that is known to potentiate the immunosuppressive functions of Tregs and thus protects tumor cells from immune attack in the hypoxic environment [47]. Notably, the hypoxia-induced transactivation of HIF-1α has been associated with an increase in the expression of metalloproteinase ADAM10 and a decrease in the surface MHC class I chain-related (MIC) levels, further highlighting the resistance of tumor cells to innate immune-mediated lysis [48,49]. HIF-1α also induces the expression of several other hypoxia-responsive genes and subsequently the production and release of chemoattractants and soluble factors, such as CSF1, TGF-β, and vascular endothelial growth factor (VEGF); these in turn regulate the differentiation and maturation of different immunosuppressive cell types, such as Tregs, TAMs, and MDSCs, at the tumor site, as well as the secretion of immunosuppressive factors, such as prostaglandin E2 and IL-10 [36,50]. 

Another pleiotropic immunosuppressive mediator that appears to be triggered by RT is adenosine, the catabolic product of adenosine triphosphate (ATP), the universal carrier of chemical energy in metabolically active cells [36]. While ATP is regarded as a key mediator of RT-induced anti-tumor immunity, known to stimulate DCs to differentiate, to process engulfed tumor antigens, and to cross-present them to naïve T cells, in the TME it is rapidly catabolized into adenosine by the actions of ectonucleotidases, mainly CD39 and CD73 [51]. Adenosine has the exact opposite effects of ATP on immunity by directly inhibiting DCs and therefore the DC-mediated activation of effector lymphocytes, as well as by promoting the proliferation and activation of immunosuppressive cell types, such as TAMs and Tregs [52,53,54]. Interestingly, both TAMs and Tregs have been found to express CD39 and CD73, and this property seems to correlate both with a sensitivity to adenosine signaling and the immunosuppressive capacity of these cells [36,55,56,57,58]. 

Last but not least, several studies have reported an upregulation of PD-L1 in the tumor micromillieu following RT, which is known to interfere with the effector functions of T cells, to assist in the immune escape of tumors and to result in treatment failure [38,59,60,61,62]. This RT-induced overexpression of PD-L1 by tumor cells can take place either via the production of IFN-γ by RT-activated T cells in immunogenic tumors, as well as in poorly immunogenic tumors where there is concomitant TGF-β blockade [11,63], or via RT-mediated upregulation of HIF-1α, as discussed above [64,65,66]. In the context of irradiated tumors, immune checkpoint blockade could represent an ideal co-therapeutic partner to RT and will be discussed into more detail in the following section.

## 5. Synergism between RT and ICIs for Optimization of Antitumor Immunity: The Need for Biomarkers

Therapeutic treatments utilizing monoclonal antibodies to block immune checkpoint molecules, the so-called immune checkpoint inhibitors (ICIs), have revolutionized the field of cancer immunotherapy by inducing remarkably durable clinical responses [67]. However, only a minority of cancer patients respond to this type of immunotherapy, thereby emphasizing the necessity for combining ICIs with other treatment modalities. The immunomodulatory mechanisms of RT, either locally or systemically, as well as the generation of potent antitumor responses by ICIs through the re-activation of exhausted T cells [67], have provided a platform for the potential synergism of RT with ICI-based immunotherapy in cancer treatment. Indeed, the combination of RT with ICIs has been shown to induce not only more potent antitumor reactivity but also concomitant impairment of tumor immune resistance mechanisms, which has in turn been associated with a significantly more favorable prognosis for overall survival. Notably, combined administration of RT with two ICIs, anti-CTLA4 and anti-PD-1, has been shown to restore vascularization within the TME, thereby not only enhancing the radiosensitivity of tumor cells, but also supporting the ability of RT to remodel the TME in favor of enhanced antitumor immunity. The latter was achieved through a variety of mechanisms in the TME, including accumulation of IFN-γ-producing CD8+ and CD4+ T cells and of eosinophils in the presence of enhanced levels of chemoattractants (CCL5, CCL11), IFN-γ, and intelrleukin-5 [68,69,70]. This suggests that the increased densities of CD4+ and CD8+ T cells, eosinophils, and the IFN-γ/CCL5/CCL11/IL-5 axis could be used as biomarkers for predicting levels of vascular normalization within the TME. The inverse correlation between hypoxia and vascularization and the negative effects of hypoxia on antitumor immune reactivity and on tumor cell radiosensitivity [68] propose that vascular improvements through immunotherapies induce TME alterations that promote sensitization to RT.

**Figure 4:**
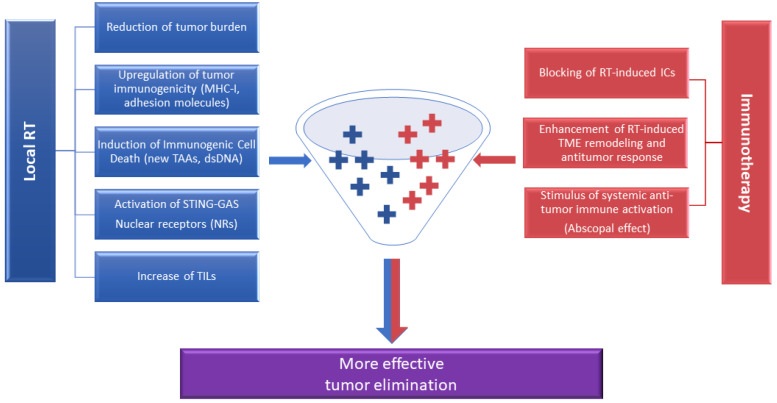
Key features underlying the synergism between radiotherapy and immunotherapy and of nuclear receptors (NRs). RT, radiotherapy; TAAs, tumor-associated antigens; MHC I, major histocompatibility complex class I; dsDNA, double-stranded DNA; TILs, tumor-infiltrating lymphocytes; ICs, immune checkpoints; TME, tumor microenvironment.

Inversely, the RT-mediated increase in PD-L1 expression levels [60,71] suggests increased response to anti-PD-1/PD-L1 therapy, further improving patient overall survival. Nonetheless, high tumor load has been reported to act as a poor predictor of clinical response to anti-PD-1 treatment, even in cases where anti-PD-1 therapy has achieved reinvigoration of T-cell-dependent antitumor immunity [72]. In such cases, the reduction of tumor mass via RT either directly, via apoptotic/necrotic death of tumor cells, or indirectly, via RT-induced antitumor cytotoxic CD8+ T cells, could further improve the clinical efficacy of anti-PD-1/PD-L1 treatment. On the other hand, PD-1/PD-L1 blockade has been shown to counteract resistance to RT. In this context, MDSCs have been shown to increase resistance of tumor cells to RT via a mechanism involving CXCL1-induced secretion of S100A8/9 proteins [71,73]. The combination of RT with PD-L1 inhibition seems to be effective in eliminating MDSCs through enhanced production of T-cell-derived TNF, thereby conferring more optimal antitumor immunity [71]. Furthermore, ICIs can boost radiation-induced abscopal response rates, and in this way generate immunologic memory and durable antitumor immunity in long-term survivors [69,74]. The link between increased RT-mediated immunogenicity and synergy with ICIs was recently shown to depend on the RT-mediated stimulation of tumor cells to produce IFN-β, a necessary prerequisite for Batf3-lineage DC activation and recruitment to the tumor site [74]. The latter facilitate cross-presentation of tumor antigens to CD8+ T cells that, in the presence of ICIs, have the ability to efficiently lyse both locally irradiated tumors and tumors at distant sites [75]. Altogether, in Figure 4, we summarize a model in which radiotherapy and immunotherapy synergistically act to promote immune activation after local RT. Although there is a plethora of reports on the activation of systemic antitumor immune responses post-RT, the issue of combining RT with other types of immunotherapy, besides ICIs, such as therapeutic cancer vaccines or cellular adoptive immunotherapy, has not been thoroughly explored. High expression of tumor antigens along with MHC class I expression is mandatory for presentation of TAAs to T cells, regardless of whether these are actively induced by a specific vaccine or exogenously introduced during T-cell-based adoptive immunotherapies. To this end, it was shown that radiation increases MHC class I expression in melanoma cell lines by expanding the intracellular tumor peptide pool, thereby increasing presentation of tumor peptides and resulting in enhanced T-cell recognition and more potent adoptive T-cell immunotherapy [31]. Thus, the effect of radiation on MHC class I expression and tumor antigen presentation may represent a useful strategy for radiation-resistant tumors, which will be sensitive to lysis either by vaccine-induced tumor peptide-specific T cells or by adoptively transferred T cells expressing receptors specifically recognizing tumor antigens. Chakraborty et al. demonstrated that local irradiation combined with a tumor vaccine in mice harboring subcutaneous tumors led to increased infiltration of the irradiated tumors by vaccine-specific CD8+ T cells, followed by tumor growth blockade [76]. Interestingly, the same study demonstrated that a significant percentage of CD8+ and CD4+ T cells in the tumor carried T-cell receptors recognizing TAAs not included in the vaccine, apparently the result of RT-enhanced TAA expression by the irradiated tumor cells. Thus, although research in this field is still evolving, we could assume that RT could function as a potent adjuvant in these types of immunotherapies.

## 6. The TCR Repertoire as a Biomarker for RT-Induced Systemic Immune Activation

All of the aforementioned studies highlight the importance of discovering biomarkers that will provide useful information as to how and to what levels RT might further potentiate the effects of ICIs when used in combination. The introduction of such biomarkers could facilitate the design of more effective treatment approaches. For example, it has been observed that the combination of ICIs with RT is more effective, in terms of producing more profound abscopal effects, when tumors are irradiated with spatial fractionation, as compared to whole irradiation [75,77]. Because abscopal effects are generated via the emergence of new T-cell clones that are cross-primed by DC loaded with tumor antigens that have been released from the radiosensitive dying tumor cells, changes in the T-cell repertoire post-RT could predict RT-induced systemic T-cell activation. 

**Figure 5:**
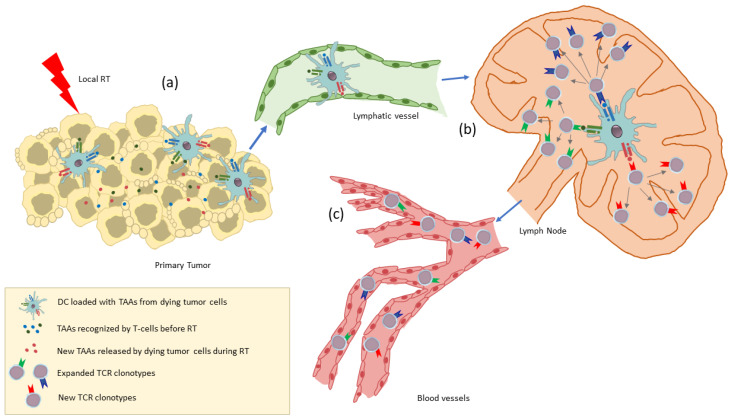
Radiotherapy leads to the release of new tumor antigens (TAAs) and thereby acts as an in situ vaccine. RT response includes release of tumor antigens (also including new TAAs) (**a**) followed by DC-mediated cross-presentation and activation of naïve T cells in the lymph node resulting in the expansion of preexisting TCR clones and in the generation of new TCR clones (**b**) that enter the circulation (**c**) and therefore can be considered as a systemic immune activation biomarker. RT, radiotherapy; TAAs, tumor-associated antigens; DC, dendritic cell; TCR, T-cell receptor.

In a very interesting study by Formenti et al., it was demonstrated that the abscopal response to the combination treatment of RT plus ICIs in patients with non-small-cell lung cancer (NSCLC) was characterized by IFN-β production and induction of systemic antitumor T-cell immunity [78]. Through the evaluation of TCR frequencies in the peripheral blood of patients, it was shown that patients who were responsive to the combination treatment had significantly higher levels of tumor-specific T-cell clones that were expanded following RT. Notably, TCR repertoire changes post-RT vs. baseline showed the highest predictive value for response to treatment compared to other variables [78]. Another study reported intratumoral T-cell clonality that was increased during RT in patients with renal cell carcinoma [79]. Interestingly, there was substantial sharing of TCR clonotypes in the tumor and blood samples at baseline. Dynamic changes to the TCR repertoire by RT were revealed by analyzing longitudinal peripheral blood samples, which showed increased frequencies of the top 10 TCR clonotypes post-RT, thus supporting the notion that radiation promotes peripheral expansion of tumor-resident T-cell clones. TCR sequencing was also performed in longitudinal blood samples from NSCLC patients receiving RT combined with anti-PD-L1 treatment; in this case, expansion of TCR clones was more frequent in treatment responders and was accompanied by a decreased TCR clonotype [80]. On the contrary, patients who clinically progressed after an initial response to therapy presented with increased TCR clonotype diversity [80]. These results demonstrate that RT may induce dynamic changes in the TCR repertoire via specific recognition of released tumor peptides from dying tumor cells resulting in systemic tumor responses. Such changes in the TCR clonal dynamic seen in cancer patients are consistent with emergence of new tumor-reactive TCR clonotypes as well as with an expansion of the tumor-directed TCR repertoire induced by RT (Figure 5).

**Figure 6:**
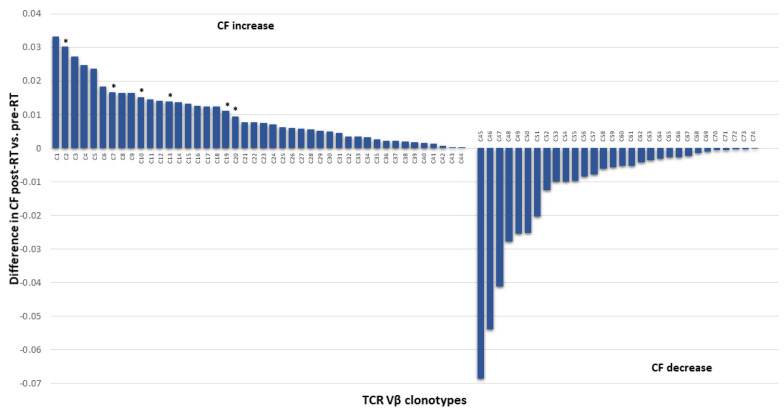
Alterations in TCR Vβ clonotypes in the peripheral blood of patients with localized prostate cancer. A number of TCR Vβ clonotypes with ascending (**left chart**) and descending (**right chart**) CFs, as well as the emergence of new clonotypes (**left chart**) with increased frequency pre- and post-RT were identified. Clonotypes with increased CFs post-RT entered the top 10 TCR Vβ CFs. Note that C2, C7, C10, C13, C19, and C20, which are presented amongst clonotypes with increased CFs, were actually new clonotypes that emerged post-RT (indicated with an asterisk). Clonotypes with decreased CFs post-RT were excluded from the top 10 TCR Vβ CFs (although they were part of the top 10 TCR CFs before RT). RT, radiotherapy; CF, clonal frequency; TCR Vβ, T-cell receptor variable β.

In our recent study, we showed for the first time TCR clonotype changes post-stereotactic body RT in patients with localized prostate cancer without any previous treatment (with the exception of two patients who underwent radical prostatectomy) [81]. In particular, clonotype frequencies (CFs) of expanded clones (including new clones) were in the range of 0.0085% to 0.033%, whereas CFs of contracted clones were in the range of 0.068% to 0.002% (Figure 6). Because of such dynamic changes post-RT, many TCR clones were accordingly included in or excluded from the top 10 TCR Vβ CFs (Figure 6). 

**Figure 7:**
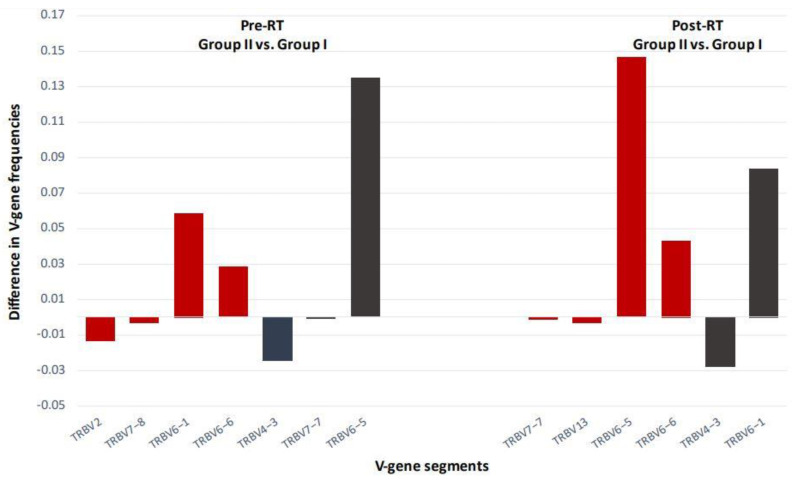
TCR V-gene usage alterations in patients with localized prostate cancer, pre- and post- RT. The graph depicts differences in the usage of specific V genes between patients with GS 8 or 7 (4 + 3) (Group II; *n* = 3) and patients with GS 6 or 7 (3 + 4) (Group I; *n* = 7). Red or black colored bars, both pre- and post-RT, correspond to V-genes with usage frequencies that differed significantly or showed strong trends between the two patient groups, respectively. TCR V-gene, T-cell receptor variable gene; RT, radiotherapy; GS, Gleason score.

Such alterations in TCR Vβ clonotypes post-RT have the potential to serve as surrogate markers of disease progression and/or response to treatment. By stratifying our patients in groups of high Gleason score (GS; Group II) vs. low GS (Group I), we were able to detect substantial changes among certain TCR V-gene segments both pre- and post-RT (Figure 7). Of note, new clones post-RT were identified among patients with high GS, suggesting that RT induces remodeling of the antitumor T-cell responses that are dependent on clinicopathological tumor characteristics (Figure 6). Our observations on T-cell expansion and contraction post-RT in patients with localized prostate cancer offer a new paradigm for the achievement of improved clinical outcomes through the combination of treatment strategies that exploit endogenous antitumor T-cell responses.

## 7. Conclusions

Immunological and cellular responses during RT act synergistically at various treatment stages to substantially decrease tumor growth rates. RT, locally or systemically, induces immunomodulatory mechanisms that sensitize T cells to tumor antigens by rendering tumors more antigenic and immunogenic, and therefore provide an important candidate for combination with immunotherapies. Nevertheless, tumor cells have integrated suppression mechanisms to evade antitumor immunity, and these may also overlap with RT resistance pathways. The discovery of biomarkers that are associated with such pathways can help to abrogate immune suppression and lead to successful therapeutic targeting. It is also imperative to identify and characterize tumor-associated antigens (e.g., mutated tumor neoantigens) that emerge post-RT as these may function as new targets for immunotherapeutic interventions. To this end, the emergence of new TCR clonotypes, and/or the dynamic alterations in the TCR post-RT, may be indicative of RT acting as an endogenous therapeutic cancer vaccine whose efficacy can be further enhanced through combination with immunotherapeutic modalities. Therefore, the precise mechanism underlying the functional role of various elements of the immune system locally in the irradiated tumors as well as in the periphery seems to be essential for the development of clinically effective therapeutic protocols. Deep sequencing of TCR CDR3 regions is emerging as a valuable method for the identification of RT-induced alterations in T-cell clonality in the peripheral blood of patients pre- and post-RT. The diversity of TCR clonotypes post-RT could potentially act as a dynamic biomarker, which could be used for the timely identification of patients that will respond to subsequent immunotherapies.
